# Exploring the Neuroprotective Properties of Celery (*Apium graveolens* Linn) Extract Against Amyloid-Beta Toxicity and Enzymes Associated with Alzheimer’s Disease

**DOI:** 10.3390/molecules30102187

**Published:** 2025-05-16

**Authors:** Layla Mohamud Dirie, Tahire Yurdakul, Sevim Isik, Shirin Tarbiat

**Affiliations:** 1 Graduate School of Science, Department of Molecular Biology, Uskudar University, 34662 Istanbul, Turkey; laylamohamuddirie.dirie@st.uskudar.edu.tr (L.M.D.); sevim.isik@uskudar.edu.tr (S.I.); 2 Stem Cell Research and Application Center (USKOKMER), Uskudar University, 34662 Istanbul, Turkey; thrarpc@gmail.com; 3 Department of Molecular Biology and Genetics, Uskudar University, 34662 Istanbul, Turkey

**Keywords:** *Apium graveolens* L., amyloid-beta, glycogen synthase kinase 3β, cholinesterase, cyclooxygenase, Alzheimer’s disease

## Abstract

Celery (*Apium graveolens* L.), one of the numerous members of the Apiaceae family, has been traditionally used as food and medicine due to its nutraceutical properties. Nevertheless, understanding the neuroprotective effects of this species requires evaluation through different mechanisms relevant to Alzheimer’s disease (AD) treatment. This study explored the neuroprotective potential of ethanolic extracts of celery leaves. Liquid chromatography and mass spectrometry-based metabolomics analysis of the extract revealed the existence of a diverse array of secondary metabolites, including phenolic acids, hydroxycinnamic acid, flavonoids, flavonoid *O*-glycosides, flavonol, glycosides, and isoflavones. Celery extract protects human neuroblastoma SH-SY5Y cells against 15 µM amyloid-beta (Aβ_1–42_) toxicity, enhancing their vitality from 67% to 81.74% at 100 µg/mL. The extract inhibited the enzymes associated with AD, including acetylcholinesterase (AChE), butyrylcholinesterase (BChE), glycogen synthase kinase 3 beta (GSK3β), cyclooxygenase 1 (COX-1), and cyclooxygenase 2 (COX-2) with IC_50_ values of 21.84, 61.27, 45.94, 34.1, and 52.2 µg/mL, respectively. In conclusion, celery leaf extract components may be potential therapeutic candidates for AD prevention and treatment.

## 1. Introduction

Alzheimer’s disease (AD) is the most prevalent neurodegenerative disease primarily affecting elderly individuals worldwide. AD is a progressive and irreversible loss of mental abilities like memory, direction, judgment, language, and the ability to learn new things. AD development is thought to be influenced by primary pathogenic pathways: amyloid-beta (Aβ) peptide deposition, tau hyperphosphorylation with forming tau neurofibrillary tangles, cholinergic deficit, oxidative stress, and chronic neuroinflammation [1,2]. Despite significant advances in understanding the pathophysiology of AD, effective therapeutic strategies remain limited.

Amyloid plaques, a hallmark of AD, are formed when Aβ peptides undergo a series of aggregation processes; the amyloid precursor protein (APP), which is extensively synthesized by brain neurons, is normally cleaved by α-secretase. Soluble monomers of Aβ peptides are produced in a non-amyloidogenic pathway, preventing plaque formation [3]. However, two consecutive proteolytic cleavages of APP by β-secretase and γ-secretase generate abnormal and Aβ peptides that tend to form oligomers. Aβ oligomers influence calcium homeostasis and disrupt synaptic function through multiple interconnected mechanisms, including the *N*-methyl-d-aspartate (NMDA) receptor dysregulation and oxidative stress. Aβ oligomers abnormally activate NMDA receptors, particularly extracellular NMDA receptors, leading to excessive calcium influx and excitotoxicity. This calcium overload impairs synaptic plasticity. Calcium overload negatively affects synaptic plasticity, diminishes long-term potentiation (LTP), and increases long-term depression (LTD), all of which contribute to cognitive decline. Concurrently, amyloid-beta (Aβ) accumulates in mitochondria, disrupting the function of the electron transport chain, which leads to an increase in reactive oxygen species (ROS) production and promotes the opening of the mitochondrial permeability transition pore (mPTP). This cascade results in further calcium dysregulation and energy failure. Moreover, the oxidative stress resulting from excessive ROS production inflicts damage on cellular components such as lipid membranes, proteins, and DNA, thereby worsening synaptic dysfunction and neuronal death. The interplay of these factors establishes a detrimental cycle of neurotoxicity, ultimately playing a significant role in the pathogenesis of Alzheimer’s disease [4,5].

The cholinergic hypothesis suggests that aging-related cognitive impairment is due to diminished cholinergic activity in the central nervous system [6]. The cholinergic system regulates neural protection, synaptic plasticity, and neuronal differentiation. Acetylcholine (ACh) is a vital neurotransmitter for healthy memory, and its hydrolysis by cholinesterase enzymes; acetylcholinesterase (AChE), and butyrylcholinesterase (BuChE) is crucial for healthy memory. Current therapeutic approaches aim to improve the cholinergic deficit by blocking AChE and enhancing cognitive functions. Three AChE inhibitors—donepezil, rivastigmine, galantamine, and memantine—are clinically licensed for Alzheimer’s disease (AD). These medications modulate neurotransmitter deficits, but they may not stop or reduce the progression of the disease [7,8]. Recent evidence suggests that AChE may be involved in plaque formation, and AChE inhibition may also retard the disease process by reducing Aβ toxicity and plaque formation [9]. Abnormal central cholinergic changes can cause abnormal tau protein phosphorylation, nerve cell inflammation, cell death, neurotransmitter imbalance, and other harmful events, though the exact mechanism is unknown [10].

Glycogen synthase kinase-3 (GSK-3) is well acknowledged as a major contributor to the onset and subsequent development of AD pathogenesis. It regulates several important targets linked to neuronal degeneration. Based on the results of the phase II study, GSK-3 has currently been considered as a potential treatment option for AD, but further proof of concept is needed. Although GSK-3β, as one of the isoforms of GSK-3, is thought to be the primary kinase responsible for tau hyperphosphorylation, it was also discovered that AD participants had significantly higher levels of peripheral and cerebral GSK-3β expression and activity, which was correlated with the development of neurofibrillary tangles. Additionally, research showed that Aβ-induced neurotoxicity can be avoided by blocking GSK-3β expression or activity. As a result, GSK-3β inhibition has been associated with decreased tau hyperphosphorylation, β-amyloid production, and neuroprotective advantages [11].

Inflammation is a key factor in the progression of various diseases, including neurodegenerative disorders like AD. Evidence that neuroinflammation contributes significantly to AD pathogenesis is still mounting. An increasing amount of evidence indicates that AD is largely caused by inflammation in the brain. Neuroinflammation in the AD brain is linked to several mechanistic pathways, including the involvement of cytokines and chemokines, the complement system, oxidative stress, and cyclooxygenase (COX) pathways [12].

Studies indicate that heightened inflammatory responses in AD-affected brain regions lead to increased levels of neuroinflammatory mediators. COXs, particularly COX-2, are significant among these. COX enzymes are upregulated in inflammatory cells and tissues. Excessive COX-2 activation in neurons contributes to increased Aβ production and cognitive decline. These findings underscore the pivotal role of COX-2 in AD pathology, emphasizing its contribution to disease progression [13].

Given the growing worldwide demand to investigate the neuroprotective properties of functional foods and their phytochemical constituents, numerous studies have highlighted the neuroprotective properties of polyphenols, flavonoids, terpenoids, and other secondary metabolites derived from plants [14]. These compounds exhibit various pharmacological actions, including antioxidants, anti-inflammatory, and enzyme-inhibitory effects, which may collectively mitigate AD-related neurodegeneration [15,16].

Celery (*Apium graveolens* L.) belongs to the Apiaceae family, widely cultivated for its edible stalks, leaves, and roots. It is native to the Mediterranean but is now grown worldwide in various climates. Celery extract’s phenolic and flavonoid contents have been investigated in many locations across the world, and the overall amounts of these compounds varied. These variations may be linked to the extraction process, environmental factors, and plant genotype. The aerial parts and roots of Egyptian celery were used to identify and characterize a variety of chemically varied metabolites, including phenylpropanoids, phthalides, coumarins, furanocoumarins, and sesquiterpenes [17,18]. Celery is a popular culinary and medicinal plant offering many nutraceutical qualities and health advantages. However, a few studies demonstrated the neuroprotective effects of Turkish native celery varieties. Our study investigates the neuroprotective potential of Turkish celery leaves, which are often overlooked despite being rich in phenolic and flavonoid compounds. Unlike other celery varieties, Turkish celery has a large root commonly used in culinary preparations. Given that environmental factors influence phytochemical composition, our research explores how regional differences impact its therapeutic properties. The novelty of this study lies in evaluating Turkish celery leaf extract against Aβ toxicity, a key factor in Alzheimer’s disease. Additionally, it is the first to examine its inhibitory effect on GSK-3β, offering new insights into its potential as an AD treatment, and in recent years, significant progress has been made in the medicinal chemistry of GSK-3β. More targeted research is still needed on this plant to highlight its molecular mechanisms and neuroprotective effects targeting multiple aspects of intricate pathogenesis. We hope that this work on amyloid-beta cytotoxicity and GSK inhibition will contribute significantly to the ongoing research on the neuroprotective benefits of celery extract as a potential therapy and preventative strategy for AD and its capacity to inhibit cyclooxygenases and cholinesterase in vitro.

## 2. Results

### 2.1. Phytochemicals Screening of Celery Leaves Extract

The phytochemical analysis is performed using a liquid chromatography-high resolution-mass spectrometry method. Fifteen phenolic compounds are tentatively identified, including phenolic acids, hydroxycinnamic acid, flavonoids, flavone, flavonoid *O*-glycosides, flavonol glycosides, and isoflavones from phytochemical classes. Large concentrations of chlorogenic acid rutin are found among them, followed by *p*-coumaric acid (Table 1 and Supplementary Figure S1).

### 2.2. Cytotoxic Effects of Aβ_1–42_ Fibrils on SH-SY5Y Cells

**Figure 1:**
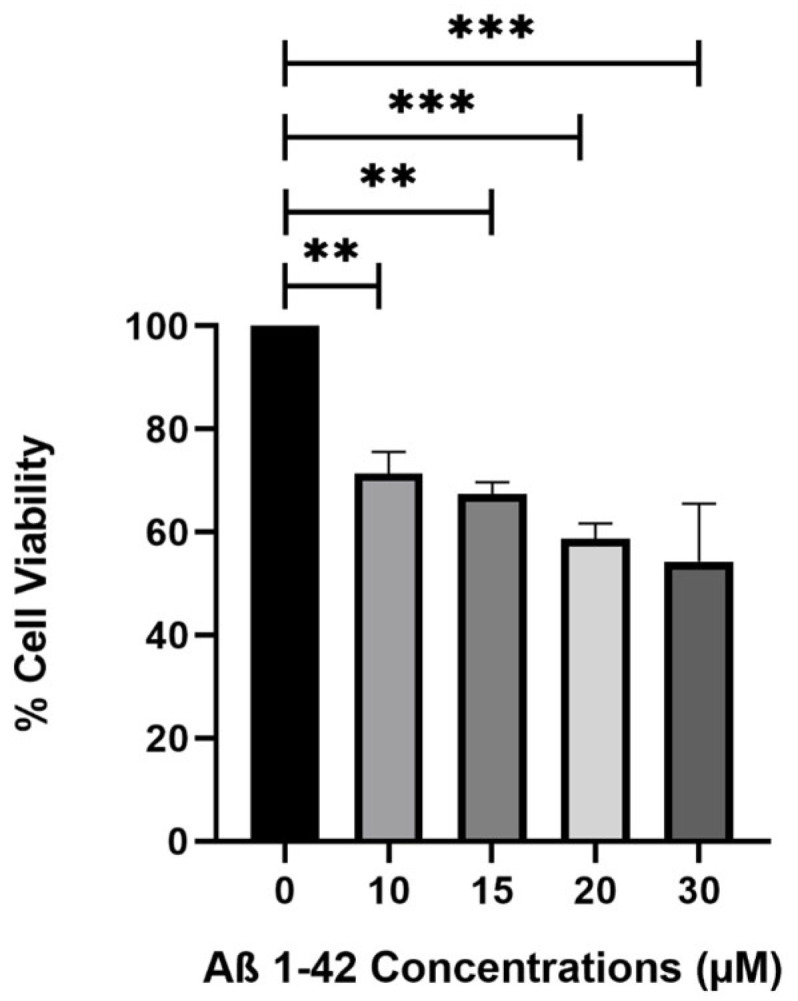
Different concentrations of 48 h of pre-incubated Aβ_1–42_ fibrils (10, 15, 20, and 30 µM) were applied to cells for 48 h. Cytotoxicity is evaluated by MTT assay, *n* = 3, and a statistically significant difference is observed between the bar pair indicated by ** (*p* < 0.05) and *** (*p* < 0.001). All groups were compared with only the control group.

The MTT test is employed to assess the cytotoxic effects of Aβ_1–42_ fibrils on SH-SY5Ys during a 48-h incubation following a 48-h pre-incubation of Aβ peptides at doses of 10, 15, 20, and 30 µM. MTT data indicate that the minimal concentration exhibiting a hazardous impact on SH-SY5Y cells is 15 µM, corresponding to 67% cell viability; subsequent tests are conducted at this concentration (Figure 1). To establish an in vitro AD model with moderate cytotoxicity (~30%), SH-SY5Y cells were treated with 15 μM Aβ_1–42_ peptide. A concentration of 10 μM (resulting in ~20% cytotoxicity) was not preferred, as it may not adequately reflect the pathological features of a moderate AD model.

### 2.3. Effect of Celery Extract on Cellular Viability

**Figure 2:**
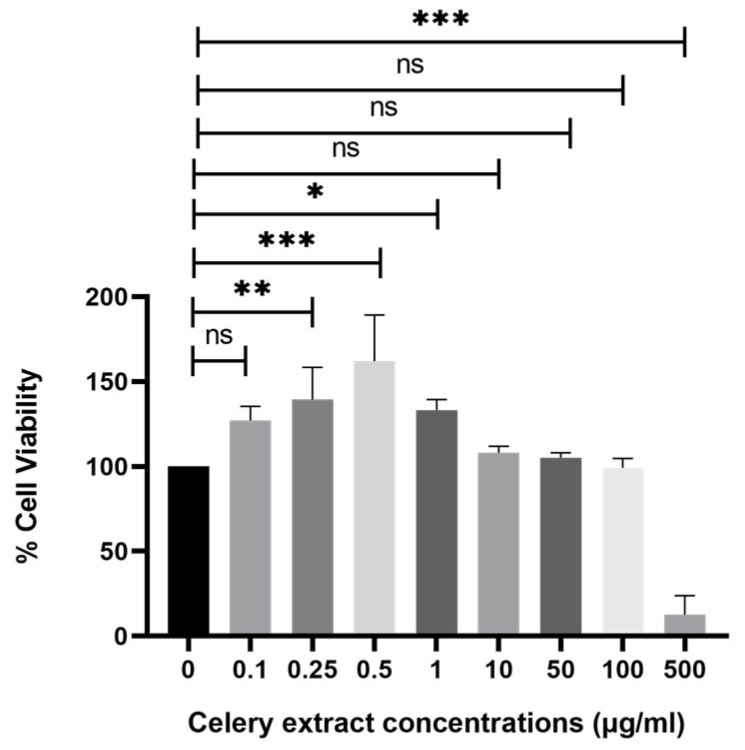
Effect of celery extract on SH-SY5Y cells’ viability. Several concentrations of celery extract (500, 100, 50, 10, 1, 0.5, 0.25, and 0.1 µg/mL) are applied to the cell culture for 48 h, and cell viability is measured. *n* = 3, a statistically significant difference is observed between the bar pair indicated by * (*p* < 0.05), ** (*p* < 0.005), and *** (*p* < 0.001). ns: nonsignificant.

The viability of SH-SY5Y cells is evaluated following treatment with various doses of celery extract. The MTT assay is employed to identify effective and safe concentrations. As illustrated in Figure 2, nearly all amounts are deemed safe, except 500 µg/mL. However, the highest three concentrations (100, 50, and 10 µg/mL), deemed safe doses, are selected to investigate the neuroprotective impact of celery extract. Their proliferative effects are indicated by cell viability values of 112%, 105%, and 101%, respectively.

### 2.4. Neuroprotective Properties of Celery Leaves Extract Against the Cytotoxicity of Aβ_1–42_ Fibrils

We have conducted an MTT experiment on SH-SY5Y cells to assess the potential neuroprotective effect of celery leaf extract against Aβ_1–42_ fibrils. Before treatment with 15 µM Aβ_1–42_, the cells undergo a 48-h pretreatment with three distinct doses of celery extract. The celery extract treatment is administered again in conjunction with the administration of Aβ.

**Figure 3:**
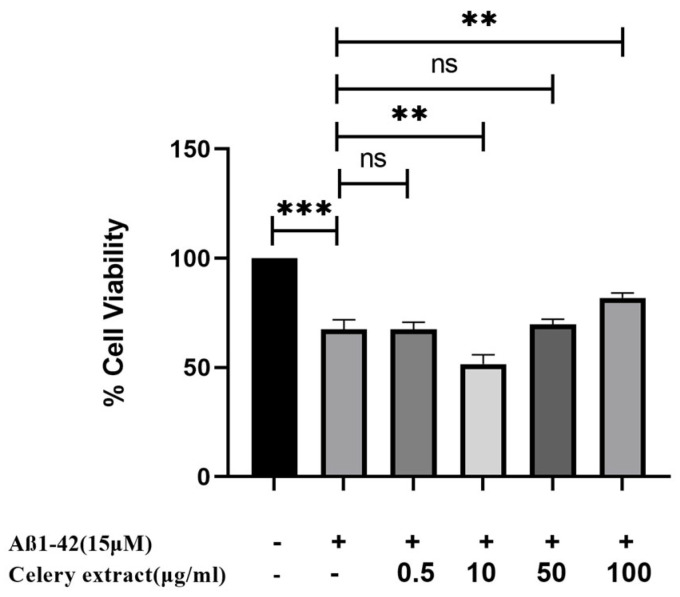
Neuroprotective effect of celery extract. SH-SY5Y cells are treated with the selected four concentrations of celery extract (0.5, 10, 50, and 100 µg/mL) for 48 h before 15 µM Aβ_1–42_ application. *n* = 3, a statistically significant difference is observed between the bar pair indicated by ** (*p* < 0.05), *** (*p* < 0.001), ns; nonsignificant.

We specifically have examined its capacity to mitigate the harmful effects induced by Aβ. The celery extract has shown considerable neuroprotection against Aβ at its maximum concentration of 100 µg/mL. This dose significantly enhances the vitality of SH-SY5Y cells from 67% to 81.74% in response to 15 µM Aβ_1–42_ toxicity compared to the Aβ control (Figure 3). However, at a concentration of 0.5 µg/mL, cell viability is 67.8%, meaning there is no effect against Aβ_1–42_ cytotoxicity. Despite its cell proliferative effect, no neuroprotective activity is observed.

### 2.5. Thioflavin S Staining to Assess Aβ_1–42_ Fibril Clearance

**Figure 4:**
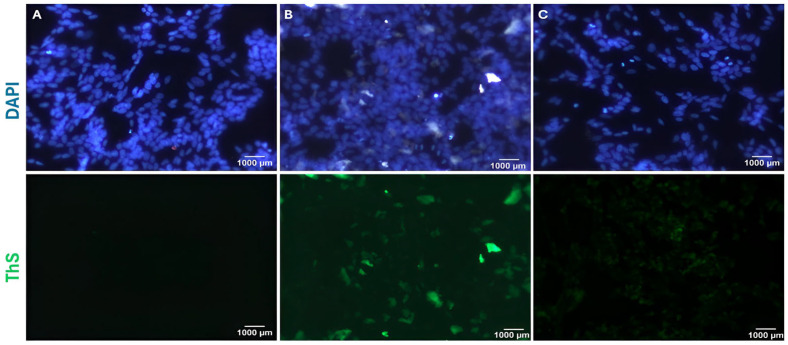
Thioflavin S staining of experimental groups. SH-SY5Y cells (**A**) are not treated, (**B**) are treated with only 15 µM Aβ_1–42_ fibrils, and (**C**) are treated with both 15 µM Aβ_1–42_ fibrils and 100 µg/mL celery leaf extract.

Thioflavin S staining is performed to evaluate the clearance of Aβ fibrils. SH-SY5Y cells are seeded into a 48-well plate. After the cells adhere to the surface, celery extract is applied at 100 µg/mL. Following 48 h of incubation, pre-incubated Aβ_1–42_ fibrils (15 µM) are added to the cells and incubated for another 48 h. The cells are then stained with 0.005% ThS, and images are acquired using a fluorescence microscope (Carl Zeiss, Axio Observer 3, Zen 3.3, Jena, Germany) (Figure 4). For this experiment, the highest dose of celery extract determined in previous studies is used.

### 2.6. Inhibition of Enzymes Associated with AD In Vitro

The inhibitory activity of celery leaf ethanolic extract against acetylcholinesterase (AChE) and butyrylcholinesterase (BChE) is evaluated using a modified Ellman’s method. For comparison, galantamine hydrobromide is used as a reference. The extract at a concentration range between (50–200 µg/mL) shows inhibitory potential against AChE. BChE inhibition is moderate compared to the reference compound. Celery extract also has shown promising GSK-3β inhibition activity with an IC_50_ value of 45 µg/mL. Staurosporine is the GSK-3β reference inhibitor. The IC_50_ values of COX enzymes indicated that the celery extract inhibits COX-1 more than COX-2 activity compared to aspirin as a reference. However, while the extract exhibits inhibitory activity against AChE, BChE, GSK-3β, COX-1, and COX-2, its potency is significantly lower than that of the standard inhibitors at (*p* < 0.05) (Table 2).

### 2.7. COX-2 Inhibition of Aβ_1–42_-Induced Neurotoxicity of SH-SY5Y Cell Lysates

**Figure 5:**
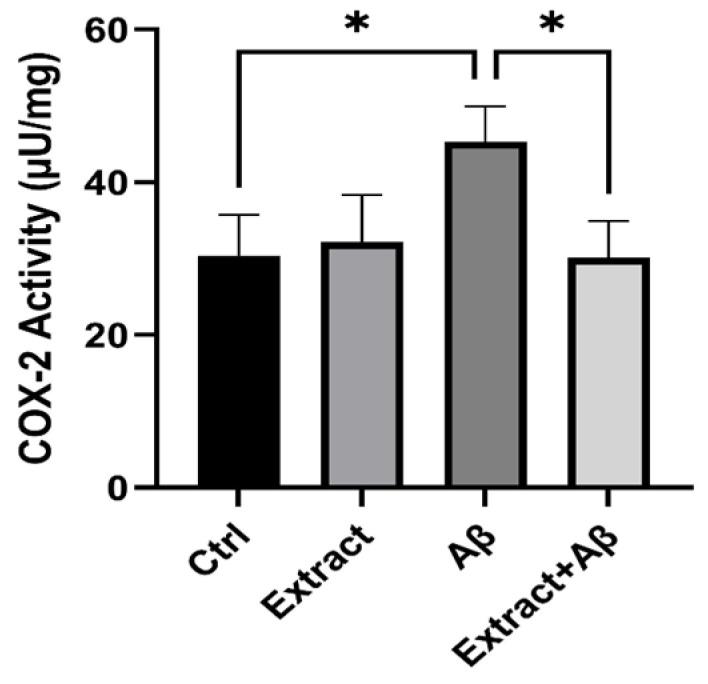
Detection of endogenous COX-2 activity (µU/mg protein) from lysates of SH-SY5Y cells in control, extract (100 µg/mL), Aβ_1–42_ (15µM), and (Aβ + extract) groups. Comparison of all groups versus each other. Differences between bar pairs marked with * (*p* < 0.05) are significant.

We also examine the endogenous COX-2 activity from lysates of Aβ-induced inflammation on SH-SY5Y cells. We observe a significant elevation of COX-2 activity in the Aβ experimental group compared to the control group (*p* < 0.05). Our results indicate that celery extract at a concentration (100 µg/mL) significantly (*p* < 0.05) protects cells from inflammation caused by Aβ toxicity (Figure 5).

## 3. Discussion

The ability of polyphenols to modulate neurotransmitter activity and reduce neuroinflammation highlights the therapeutic potential of the plant’s secondary metabolites in improving cognitive function and potentially slowing down the progression of this neurodegenerative disorder. Understanding the mechanism of their action and long-term effects is crucial for fully understanding their therapeutic role in Alzheimer’s disease (AD) management [19].

The in vitro, pre-clinical, and clinical studies examined the therapeutic role of phytochemicals in neuroprotection against AD; for instance, plants Panax ginseng, *Ginkgo biloba*, *Bacopa monnieri*, *Withania somnifera*, Curcuma longa, and *Lavandula angustifolia* have potential for treating AD. The plants’ active components and neuroprotective properties were studied, and their compounds showed therapeutic effects by reversing neurological changes; improving concentration, memory, cognition, and learning; and reducing interleukin levels and tumor necrosis factor alpha [20].

In the present study to confirm the neuroprotective effect of celery extract, the cytotoxic effects of Aβ_1–42_ fibrils on SH-SY5Y cells and the neuroprotective effect of celery extract against this toxicity were evaluated. The results demonstrate that Aβ_1–42_ fibrils significantly decrease cell viability at specific concentrations, while celery extract has the potential to mitigate these negative effects. Aβ_1–42_, a prominent peptide in AD, forms fibrils that induce neurotoxicity and mimic the cellular damage observed in the disease. Treatment of SH-SY5Y cells with Aβ_1–42_ fibrils has been established as a viable model for investigating the pathogenesis of AD and is essential for comprehending the processes of neurotoxicity and assessing potential therapeutic agents that aim to reduce Aβ_1–42_-induced neuronal damage [21].

Upon treatment with different concentrations of celery extract (0.5, 10, 50, 100, and 500 µg/mL) for 48 h before Aβ_1–42_ application, cell viability showed a dose-dependent improvement. The lowest concentration (0.5 µg/mL) does not seem to provide a significant protective effect, as cell viability remains similar to Aβ_1–42_ treatment alone. However, at higher concentrations (10, 50, and 100 µg/mL), cell viability progressively increases, with the 100 µg/mL treatment showing the most pronounced protective effect. More precisely, the administration of higher concentrations of celery extract was associated with a more significant neuroprotective effect, which is consistent with prior research indicating that the level of neuroprotection provided by phytochemicals from plant extracts might vary depending on their concentration [22]. On the other hand, 500 µg/mL demonstrated cytotoxic effects on the cells. However, at 500 µg/mL, cytotoxicity is induced, indicating that celery extract becomes cytotoxic to cells above a particular threshold. There could be multiple reasons for this hazardous impact at 500 µg/mL, such as overstimulation of signaling pathway that causes apoptosis, disruptions in calcium signaling pathways, or formation of excess free radicals. Consequently, even though celery extract exhibits neuroprotective effects between 10 and 100 µg/mL, its cytotoxicity at 500 µg/mL emphasizes the necessity of cautious dosing to optimize therapeutic benefit while preventing side effects. To ensure the safe and effective use of the extract in neuroprotective applications, future research should focus on investigations in vivo studies to determine therapeutic dosages and to clarify the mechanisms of toxicity at high concentrations.

Peng et al. [23] investigated the neuroprotective effects of L-3-*n*-butylphthalide produced from seeds of *Apium graveolens* Linn, a native species of China, using a triple-transgenic-AD mice model, which results in plaques and tangles with age and cognitive impairments. According to their research, L-3-*n*-butylphthalide treatment considerably improved spatial memory and learning deficits in transgenic AD mice. Furthermore, it decreased the deposition of total cerebral Aβ plaque and increased the release of soluble amyloid-beta precursor protein (APP), indicating that this bioactive from celery seeds may prevent the synthesis of Aβ and redirect APP processing to a non-amyloidogenic pathway.

AD is characterized by elevated levels of the inducible enzyme cycloxygenase-2 (COX-2), which is linked to inflammation and oxidative damage. Overexpression of COX-2 increases neuroinflammation by promoting tau hyperphosphorylation, Aβ aggregation, and neuronal damage; while COX-1 promotes homeostatic processes, it also promotes neuroinflammation. The advantages of employing COX-2 and COX-1 selective inhibitors as a treatment strategy for neurodegenerative diseases are significant [24]. In the present study, celery leaf extract has been examined in vitro for COX-1 and COX-2 inhibitory effects. Celery extract has shown high inhibitory potential on COX-2 that was comparable to aspirin as a non-steroidal anti-inflammatory drug (NSAID). The development of AD is also supported by several epidemiological studies that demonstrate that using NSAIDs can reduce the risk of developing AD [25]. Reports suggest that anti-inflammatory therapies were effective in altering Aβ processing and deposition [26]. As a result, neuroinflammation plays an important role in AD development. Shine et al. [27] examined the anti-inflammatory properties of hydrolyzed celery leaf extract and demonstrated a noteworthy decrease in COX-2 mRNA expression in primary splenocytes activated by concanavalin A.

In the present study, we also examine the endogenous COX-2 activity from lysates of Aβ-induced inflammation on SH-SY5Y cells. In neuronal cell lines such as SH-SY5Y, inflammation-related responses typically refer to the activation of pro-inflammatory pathways, particularly through the upregulation of enzymes and mediators involved in neuroinflammation, such as cyclooxygenase-2 (COX-2), or the release of pro-inflammatory cytokines triggered by neurotoxic agents like amyloid-beta (Aβ_1–42_). We observe a significant elevation of COX-2 activity in the Aβ experimental group. Prostaglandins, produced by COXs, have been linked to the development of inflammation [28]. The celery extract significantly inhibits the formation of endogenous prostaglandin in Aβ-induced inflammation in SH-SY5Y cells (Figure 5). Our findings suggest that one potential mechanism by which celery extract could inhibit inflammatory processes in AD is by inhibiting COX-2 activity.

Acetylcholinesterase (AChE) and butyrylcholinesterase (BChE) are two key cholinesterases. Increased cholinesterase activity worsens cognitive decline by reducing synaptic acetylcholine (ACh) levels; therefore, inhibiting cholinesterase activity is an effective strategy to control synaptic dysfunction, thereby increasing ACh levels [29]. According to the experimental results displayed in Table 2, the celery extract exhibited a strong inhibitory effect on AChE. Boga et al. [30] investigated the dichloromethane, ethanol, and water extracts of the roots of celery from Turkey, and they found that the dichloromethane, ethanol, and water extracts inhibited BChE by 59.9%, 40.39%, and 26.73%, respectively in 200 µg/mL, while the extracts have not shown inhibitory effect at the same concentration on AChE activity.

Cholinergic dysfunction is brought on by neuroinflammation. COX-2-driven inflammation leads to oxidative stress and cytokine release (IL-1β, IL-6, and TNF-α), enhancing AChE and BChE activity. Accordingly, COX-2 inhibition may reduce neuroinflammation and potentially protect cholinergic neurons. Additionally, elevated AChE activity aggravates neurodegeneration by promoting Aβ aggregation. Aβ-induced neuroinflammation further activates COX-2, creating a vicious cycle of neuroinflammation and cholinergic dysfunction [31,32,33]. Thus, inhibiting cholinesterase and COX enzymes by celery extract components provides synergic benefits through its polyphenols and phytocomponents, which could offer neuroprotection by reducing inflammation and enhancing cholinergic signaling.

Glycogen synthase kinase (GSK-3β) is another important enzyme associated with AD development and progress. Both natural and synthetic GSK-3β inhibitors were reported to reduce tau hyperphosphorylation and Aβ formation, decreasing the production of inflammatory chemicals and oxidative stress in the brain [11]. Our findings imply that celery extract’s GSK3 inhibitory activity may be mediated by its phenolic-rich components, which also protect against amyloid-beta toxicity [34]. A well-known example is curcumin, a polyphenol from turmeric, which has been extensively studied for its neuroprotective effects. Curcumin’s effectiveness in rats with scopolamine-induced AD was evaluated. The results revealed a reduction in the amount of phosphorylated tau protein and Aβ in the brain and plasma. Additionally, curcumin decreased total GSK3β, phospho-GSK3β, and the active version of GSK3β, which may have reduced tau hyperphosphorylation and Aβ aggregation [35].

There are no clinical trials currently investigating the effects of *Apium graveolens* (celery) on Alzheimer’s disease. Pre-clinical studies have shown that 3-*n*-butylphthalide (NBP), a bioactive compound found in celery, has neuroprotective properties. NBP has shown efficacy in animal models, improving cognitive impairment and reducing amyloid-beta levels. Further research is needed to determine the potential therapeutic role of *Apium graveolens* or its constituents in Alzheimer’s treatment. In the field of neurodegenerative disorders, only one clinical trial involving celery and its active ingredient, DL-NBP, showed improvements in symptoms like bradykinesia, stiffness, sleep quality, and quality of life in patients with Parkinson’s disease [36].

Celery extract contains phenolic and flavonoid compounds with potential neuroprotective effects. While some of its compounds, like 3-*n*-butylphthalide, can cross the blood–brain barrier, their bioavailability and metabolic stability need to be considered. Future research could use nanocarriers or chemical modifications to enhance the CNS accessibility of celery-derived compounds, potentially making them more effective for treating neurodegenerative diseases like Alzheimer’s [37].

While the neuroprotective effects of celery leaf extract, as evidenced by its inhibitory effects on AChE, BChE, amyloid-beta aggregation, and COX-2, suggest promising benefits, the absence of bioavailability and pharmacokinetic data represents a significant limitation. Flavonoids and polyphenolic compounds, commonly found in such extracts, are known to exhibit low oral absorption and undergo rapid metabolism, which can substantially affect their efficacy in vivo. Therefore, future pharmacokinetic studies are crucial to assess the stability, absorption, distribution, metabolism, and excretion (ADME) profile of celery leaf extract components to fully understand their impact and optimize their clinical application.

## 4. Materials and Methods

### 4.1. Materials

GSK-3β Kinase Enzyme System kit (catalog number: V1991) was purchased from Promega GmbH, Walldorf, Germany. Fluorometric COX Assay Kit (ab204699) was obtained from Sigma-Aldrich (St. Louis, MO, USA), originally developed by Abcam (Waltham, MA, USA). The other chemicals were acquired from Sigma-Aldrich Co. (St. Louis, MO, USA). SH-SY5Y (human neuroblastoma) cell lines were used in this investigation. The American Type Culture Collection (ATCC, Manassas, VA, USA) provided the SH-SY5Y cell line (CRL-2266).

#### Plant Material

*Apium graveolens* Linn (celery) was collected in April 2024 at full maturity (approximately 3 months old, typical maturity for harvesting celery leaves for phytochemical analysis) from an agricultural company located in Antalya, Turkiye (Antmek Tarım Ürünleri Gıda San. ve Tic. Ltd. Şti., Antalya, Turkey).

### 4.2. Celery Extract Preparation

The plant material following harvest was thoroughly washed and rinsed with distilled water and then gently dried in the shade at 24–26 °C, and the leaves were removed from the roots and aerial parts. The celery leaf powder (10 g), which was air-dried, was extracted using 300 mL of 95% ethanol at 60 °C for 6 h in a Soxhlet extractor (Buchi Universal Extraction System B-811, Essen, Germany). The ethanol used to obtain the extract was removed using a rotary evaporator at 40 °C under reduced pressure (approximately 100–150 mbar) until a concentrated extract was obtained. The dried extract was stored in amber glass vials with tightly sealed screw caps at 4 °C, protected from light. The extract yield was 0.478 g (4.78%) and stored at +4 °C for future use.

### 4.3. LC-HR-MS Analysis of Extracts

Chromatographic separation was carried out using an Agilent 1260 Infinity II HPLC system coupled to an Agilent 6540B Q-ToF mass spectrometer (Santa Clara, CA, USA). A Troyasil C18 HS column (İstanbul, Turkey, 150 × 3 mm, 3.5 µm particle size) was used and maintained at a column oven temperature of 40 °C. The injection volume was 5 µL. The mobile phases were solvent A (1% formic acid in HPLC-grade water) and solvent B (1% formic acid in HPLC-grade methanol). All solvents were of LC-MS grade (≥99.9% purity). A linear gradient elution was employed as follows: 50% B (0–3 min), increasing to 100% B (3–6 min), held at 100% B (6–7 min), and returned to 50% B from 7–15 min. The flow rate was 0.35 mL/min. Mass spectrometric detection was performed with a Dual AJS ESI source in positive ionization mode (Agilent). The ESI source parameters were as follows: drying gas temperature 325 °C, drying gas flow 10 L/min, nebulizer pressure 35 psi, sheath gas temperature 350 °C, sheath gas flow 11 L/min, and capillary voltage 3500 V. Fragmentation was performed using data-dependent MS/MS acquisition mode, with collision energy set according to a ramped protocol based on the m/z range of analytes. The ethanolic extract was prepared at a concentration of 0.5 mg/mL, centrifuged at approximately 13,000× *g* for 10 min at 4 °C, and filtered through a 0.22 µm PTFE syringe filter (Thermo Fisher Scientific, Waltham, MA, USA). Data acquisition was conducted in both MS and MS/MS modes. Phytochemical identification and quantification were based on external standards, with compound identity confirmed by matching retention time (RT), accurate *m*/*z*, and fragmentation patterns to authentic standards, in accordance with confidence level 1 of Schymanski et al. [38].

### 4.4. Culture of SH-SY5Y Cells

The SH-SY5Y cell line, originating from human neuroblastoma, was acquired from the American Type Culture Collection (ATCC). The cells were cultured in Dulbecco’s Modified Eagle’s Medium (DMEM) according to ATCC instructions, supplemented with 15% (*v*/*v*) fetal bovine serum (FBS), 1% (*v*/*v*) L-glutamine, 1% (*v*/*v*) sodium pyruvate, and 1% penicillin/streptomycin. The culture was maintained at 37 °C with a 5% CO_2_ concentration in a 95% humidified incubator. Medium refreshment was conducted every 48 h, and subculture was repeated weekly. To separate the SH-SY5Y cells from 25 cm^2^ culture flask, 1 mL 0.25% trypsin/EDTA solution (Gibco cat. no: 25200056, Waltham, MA, USA) was used upon reaching 70% confluency. The cells were inoculated in a fresh culture flask at a 4 × 10^4^ cells/cm^2^ density.

### 4.5. In Vitro Alzheimer’s Disease Model

The toxic dose of Aβ_1–42_ fibrils was determined following a 48-h incubation period. Amyloid-beta peptide (1–42) (human, >95% purity, Abcam, cat no: ab120301) was dissolved in 1% (*v*/*v*) NH_4_OH/dH_2_O and then diluted with 1X PBS 1X D-PBS (Ca & Mg (WISENT MULTICELL, Saint-Jean-Baptiste, QC, Canada, cat no: 311-425 CL) to achieve a final 1 mg/mL concentration. Fibril formation was induced by incubating the peptide solutions in 1.5 mL microcentrifuge tubes at concentrations of 10, 15, 20, and 30 µM at 37 °C, 5% CO_2_ for 48 h with gentle agitation in a 95% humidified incubator. SH-SY5Y cells were seeded at a density of 3 × 10^4^ cells in 100 µL DMEM medium per well in a polystyrene, round well with flat-bottom 96-well plate with 300 µL maximum volume. Afterward, the cells were treated with pre-formed Aβ_1–42_ fibrils for an additional 48 h incubation at 37 °C, 5% CO_2_ and 95% relative humidity [39]. Cell viability was assessed using the MTT reagent (3-[4,5-dimethylthiazol-2-yl]-2,5-diphenyltetrazolium bromide, Roche cat no: 11465007001, Basel, Switzerland) following the manufacturer’s protocol. Absorbance readings were taken at 570 nm using an advanced filter-based multi-mode microplate reader (BMG LABTECH, FLUOstar Omega, Offenburg, Germany).

### 4.6. Establishing the Optimal Dose of Celery Leaf Extract and Its Use on SH-SY5Y Cells

The celery extract was dissolved in DMSO at 50 mg/mL. SH-SY5Y cells were seeded in a polystyrene, round well with flat-bottom 96-well plate at a concentration of 10^4^ cells per well in 100 µL complete DMEM medium (15% (*v*/*v*) FBS, 1% (*v*/*v*) l-glutamine, 1% (*v*/*v*) sodium pyruvate, and 1% (*v*/*v*) pen/strep) per well in triplicate, and the cells were then grown for a full day at 37˚C, 5% CO_2_ and 95% relative humidity. The stock extract solution was diluted with complete cell culture medium to achieve a final DMSO concentration of 0.1% and then given to the cells at concentrations of 500, 100, 10, 1, 0.5, 0.25, 0.1, and 0.01 μg/mL for 48 h at 37 °C, 5% CO_2_ and 95% humidified incubator to determine the optimal dose. After that, the MTT cell proliferation and cytotoxicity test kit (Roche, cat no: 11465007001) was used according to the instructions provided by the manufacturer. The 110 µL MTT reagent (final concentration 0.5 mg/mL) was then added and incubated for 4 h without light. After adding 100 μL of solubilization buffer to make a total volume of 210 µL to the MTT solution in each plate well, the mixture was incubated for an entire night at 37 °C and 5% CO_2_ in 95% humidified incubator until the formazan crystals were completely dissolved. An automated microplate analyzer advanced filter-based multi-mode microplate reader (BMG LABTECH, FLUOstar Omega) assessed the absorbance at a wavelength of 570 nm.

### 4.7. Celery Leaf Extract Treatment Against Aβ_1–42_ Fibril Cytotoxicity

SH-SY5Y cells were cultivated for 24 h in a polystyrene, round well with flat-bottom 96-well plate at a density of 10^4^ cells in 100 µL complete DMEM medium per well at 37 °C and 5% CO_2_ in a 95% humidified incubator. To evaluate the neuroprotective effects of celery extract against Aβ_1–42_-induced cytotoxicity, cells were treated with three distinct doses of celery extract in complete DMEM medium at 37 °C and 5% CO_2_ for 48 h before the introduction of Aβ_1–42_ fibrils. The maximum non-toxic dilutions of celery extract selected for this experiment are 50, 100, and 500 µg/µL. Cells were grown for 48 h following treatment of each cell with 15 μM of Aβ and extract with the exact dose previously administered. The positive control was the AD group, which was treated with only Aβ fibrils, and the negative control was the untreated group. The MTT test was utilized to determine cell growth.

### 4.8. Thioflavin S Staining

Cells were inoculated at a density of 4.5 × 10^4^ cells per well in 200 µL complete DMEM medium in a flat-bottom 48-well plate (Greiner, Kremsmunster, Austria). Upon completion of the Aβ_1–42_ fibril incubation, cells were subjected to a 4% paraformaldehyde/phosphate buffer saline (PFA/PBS) solution for fixation for 10 min at room temperature, followed by a 5-min wash with PBS (300 µL/well) at 30 rpm on linear shaker. Subsequently, Thioflavin S powder (ThS) (Sigma, St. Louis, MO, USA, T1892) was dissolved in EtOH/dH_2_O (1:1) solution to prepare 0.025% ThS solution and diluted to 0.005% with dH_2_O, and the cells were incubated for 10 min at room temperature. Because of this substance’s sensitivity to light, work was done in complete darkness at the time. Upon completion of this incubation, cells were rinsed with 70% EtOH (500 µL/well) for 5 min at 30 rpm on linear shaker repeated this process three times; thereafter, cells were incubated in 1:5000 dilution of 4′,6-diamidino-2-phenylindole (DAPI), which was dissolved in dH_2_O for 5 min for nuclear staining. The cells were then washed once with dH_2_O and once with PBS, respectively [40].

### 4.9. Cholinesterase Inhibition

AChE and BuChE inhibitory effects were performed using Ellman’s method. A mixture comprising 200 μL of an AChE/BuChE solution (0.415 U·mL^−1^ in 0.1 M phosphate buffer, pH 8.0), 100 µL of a DTNB (5,5′-dithiobis-(2-nitrobenzoic acid) solution (3.3 mM in 0.1 M phosphate-buffered solution, pH 7.0) containing NaHCO_3_ (6 mM), varying concentrations of celery leaf extract (50–200 µg/mL), and 500 µL of phosphate buffer, pH 8.0, were used to measure the extract’s inhibitory activity. After 20 min of incubation at 25 °C, the substrate—100 µL of a 0.1 mM acetylcholine iodide/butyryl thiocholine chloride solution—was added to the cuvette. Using a spectrophotometer (UV-2600, Shimadzu, Kyoto, Japan), the change in absorbance at 412 nm for 3 min at 25 °C was used to calculate the AChE/BuChE activity [41]. AChE and BChE enzymes were isolated from the brains of freshly sacrificed, healthy adult Wistar rats. Brain tissues were homogenized in ice-cold 0.1 M phosphate buffer (pH 7.4) using a glass-Teflon homogenizer, followed by centrifugation at 10,000× *g* for 15 min at 4 °C. The supernatant was collected and used immediately as the crude enzyme source in the inhibition assays.

In both tests, galantamine hydrobromide served as a reference compound. The concentration ranges were 0.01 to 1 and 1 to 20 (µg/m) for AChE and BChE, respectively. The celery extract IC_50_ values were determined against AChE and BChE in a concentration range between 50–200 µg/mL using GraphPad Prism 9.5.1.

### 4.10. GSK-3β Inhibition Assay

The inhibition assay for glycogen synthase kinase-3β was performed according to the kit instructions (Liu et al., 2021 [42]). Kinase buffer was used to dilute the enzyme, substrate, ATP, and inhibitor. Then, 1 µL of extract (50–200 µg/mL) was diluted to the appropriate concentration in the assay buffer. After 2 µL of enzyme and 2 µL of substrate/ATP were mixed, the solution was incubated for 60 min at room temperature. Later, the enzyme reaction was halted using 5 µL of ADP-GloTM Reagent termination reagent, and it was then allowed to sit at room temperature for 40 min. After that, 10 µL of kinase detection reagent was used to convert ADP to ATP for 30 min. Finally, the luminous value was recorded using a multipurpose microplate reader (BMG LABTECH, FlouOstar Omega) [43]. The values are calculated by GraphPad Prism software version 9.5.1. Staurosporine served as a reference.

### 4.11. COX-1 and COX-2 Inhibition

Cyclooxygenase (COX) is a membrane-bound enzyme that converts arachidonic acid to prostaglandin G (PGG) and reduces PGG to PGH, which is the reduced product of prostaglandin G, leading to the production of prostanoids. COX is expressed in two isoforms: constitutively expressed COX-1 and inducible COX-2. Inflammation mediators like growth factors, cytokines, and endotoxins cause COX-2 production in various biological systems.

COX-1 and COX-2 inhibitory activities of celery leaf extract at a concentration of 50–200 µg/mL were determined in vitro. The arachidonic acid solution is added as a substrate into each well to start the reaction after the standard curve and reaction mixture are prepared according to the kit instructions. Fluorescence measurement was done in a kinetic mode at room temperature (25 °C) for 30 min, with an excitation/emission ratio of 535/587 nm (BMG LABTECH, FlouOstar Omega). The quantity of cyclooxygenase that produces 1.0 µmol of resorufin per minute at pH 8.0 and 25 °C was equivalent to one unit of COX activity. The resorufin formation was the basis for quantification, using the resorufin standard provided in the kit. The standard curve according to the kit procedure was prepared. Aspirin served as a reference substance.

### 4.12. Total Protein Determination

Prior to protein collection, SH-SY5Y cells were seeded in a six-well plate (Greiner) at a density of 4 × 10^5^ cells/well in complete DMEM medium, and they were grown at 37 °C and 5% CO_2_ in a 95% humidified incubator for 24 h. After that, cells were treated with 1.5 mL celery extract (100 µg/mL) at 37 °C and 5% CO_2_ in a 95% humidified incubator for 48 h. Pre-treated SH-SY5Y cells were exposed to 48-h pre-incubated Aβ_1–42_ fibrils for another 48 h with the exact dose of celery extract (100 µg/mL). The protein was isolated while experimenting on ice. Firstly, the old medium was discarded from the six-well plate (Greiner), and each well was rinsed with 1.5 mL of cold PBS on ice. Subsequently, RIPA buffer (80 µL) (Serva, Heidelberg, Germany, cat no: 39244.01) comprising 1% (*v*/*v*) SDS and 1% (*v*/*v*) protease inhibitor cocktail was added to lyse the cells. The scraper (20 mm) (Nest, Wuxi, China, cat no: 710011) was promptly employed to meticulously scrape the wells, and the harvested cells in 80 µL RIPA buffer were thereafter transferred into a 1.5 mL centrifuge tube. The samples were homogenized in 1.5 mL centrifuge tube with the MagNA Lyser (Roche) without beads at 6000 rpm for 15 s, repeated twice. The samples were transferred and incubated on ice in between runs for 2 min to cool down. Then, samples were subsequently heated at 95 °C for 2 min, and the protein specimens were used immediately for COX-2 assay. The Lowry method was used to determine the total protein level [44].

### 4.13. COX-2 Inhibitory Potential of Extract on Aβ_1–42_-Induced Neurotoxicity of SH-SY5Y Cell Lysates

The cycloxygenase-2 (COX-2) activity was determined from (15 µM) Aβ-induced toxicity and pre-treated celery extract (100 µg/mL) of Aβ-induced toxicity of SH-SY5Y cell lysates. The arachidonic acid solution is added as a substrate into each well to start the reaction after the standard curve and reaction mixture are prepared according to the kit instructions. Fluorescence measurement was completed in kinetic mode at room temperature for 30 min, with an excitation/emission ratio of 535/587 nm. The quantity of cyclooxygenase that produces 1.0 µmole of resorufin per minute at pH 8.0 and 25 °C was equivalent to one unit of COX activity.

### 4.14. Statistical Analysis

The mean ± standard deviation is used to represent all data. Multi-group comparisons were performed using one-way ANOVA using GraphPad Prism software version 9.5.1.

## 5. Conclusions

In conclusion, there remains considerable scope for further investigation into the phytochemical composition of celery leaf extract and its relevance to Alzheimer’s disease (AD) prevention and treatment. Our findings demonstrate that the extract exhibits inhibitory effects on key targets involved in AD pathology, including GSK-3, COX enzymes, and cholinesterases, suggesting a promising multi-target therapeutic approach. Given the chemical complexity of celery, future studies should focus on elucidating the individual and synergistic effects of its phenolic compounds. Extensive in vitro studies are warranted to define the specific molecular interactions between these compounds and targets such as COX-2 and GSK-3β, while in vivo research should explore their pharmacokinetics, bioavailability, and neuroprotective potential in AD models.

## Figures and Tables

**Table 1:** Phytochemicals screening of ethanolic extract of celery leaves using LC-HRMS.

Phytochemicals Class	Compounds	mg/g of Extract	Relative Uncertainty (%)
Phenolic acid	Chlorogenic acid	0.637	3.58
Phenolic acid	Fumaric acid	0.056	2.88
Phenolic acid	Caffeic acid	0.0039	3.74
Flavone	Luteolin-7-rutinoside	0.0098	3.06
Phenolic acid	Vanillic acid	0.0351	3.49
Phenolic acid	Sinapinic acid	0.0695	3.57
Flavonoid *O*-glycosides	Luteolin-7-glucoside	0.0410	4.14
Hydroxycinnamic acid	*p*-Coumaric acid	0.1288	3.31
Flavonoid glycoside	Rutin	0.4944	3.07
Flavonol glycoside	Hyperoside	0.0027	3.46
Flavonoid	Apigenin-7-glucoside	0.0129	3.59
Flavonoid	Nepetin-7-glucoside	0.0002	3.07
Phenolic acid	Salicylic acid	0.0029	1.89
Flavanone	Naringenin	0.0016	4.20
Isoflavone	Genistein	0.0044	3.28

**Table 2:** Inhibition of AChE/BuChE, GSK-3b, and COX-1/COX-2 by celery (*Apium graveolens* L.) leaf extract in vitro.

	AChE IC_50_ (µg/mL)	BChE IC_50_ (µg/mL)	GSK-3β IC_50_ (µg/mL)	COX-1 IC_50_ (µg/mL)	COX-2 IC_50_ (µg/mL)
Extract	21 ± 1.1 *	61 ± 1.1 **	45 ± 1.1 **	34 ± 1.1 **	52 ± 1.2 *
Galantamine hydrobromide	0.078 ± 1.2	5.6 ± 1.1	-	-	-
Staurosporine	-	-	0.02 ± 1	-	-
Aspirin	-	-	-	4.4 ± 1.2	30 ± 1

Note: Statistical significance was determined using an independent *t*-test (Welch’s *t*-test assuming unequal variances). ** *p* < 0.05, * *p*< 0.001 indicate statistically significant differences between the extract and the positive control.

## Data Availability

The data are available from the corresponding author upon suitable request.

## Supplementary Materials

The following supporting information can be downloaded at: https://www.mdpi.com/article/10.3390/molecules30102187/s1, Figure S1. (a–c); The chromatogram from Phytochemicals’ LC-MS (Liquid Chromatography-Mass Spectrometry) analysis of the ethanolic leaf extract of celery (Table 1) shows multiple extracted ion chromatograms (EICs) with different retention times (RT) and corresponding mass-to-charge ratios (*m*/*z*).
