# Supplementary material for: Exploring the Neuroprotective Properties of Celery (Apium graveolens Linn) Extract Against Amyloid-Beta Toxicity and Enzymes Associated with Alzheimer’s Disease
Source: Molecules. 2025 May 16;30(10):2187. doi: 10.3390/molecules30102187 (PMC12113848; doi:10.3390/molecules30102187)
Supplement: Supplementary file 1 [file molecules-30-02187-s001.zip › molecules-3550031-supplementary.pdf]

(a)

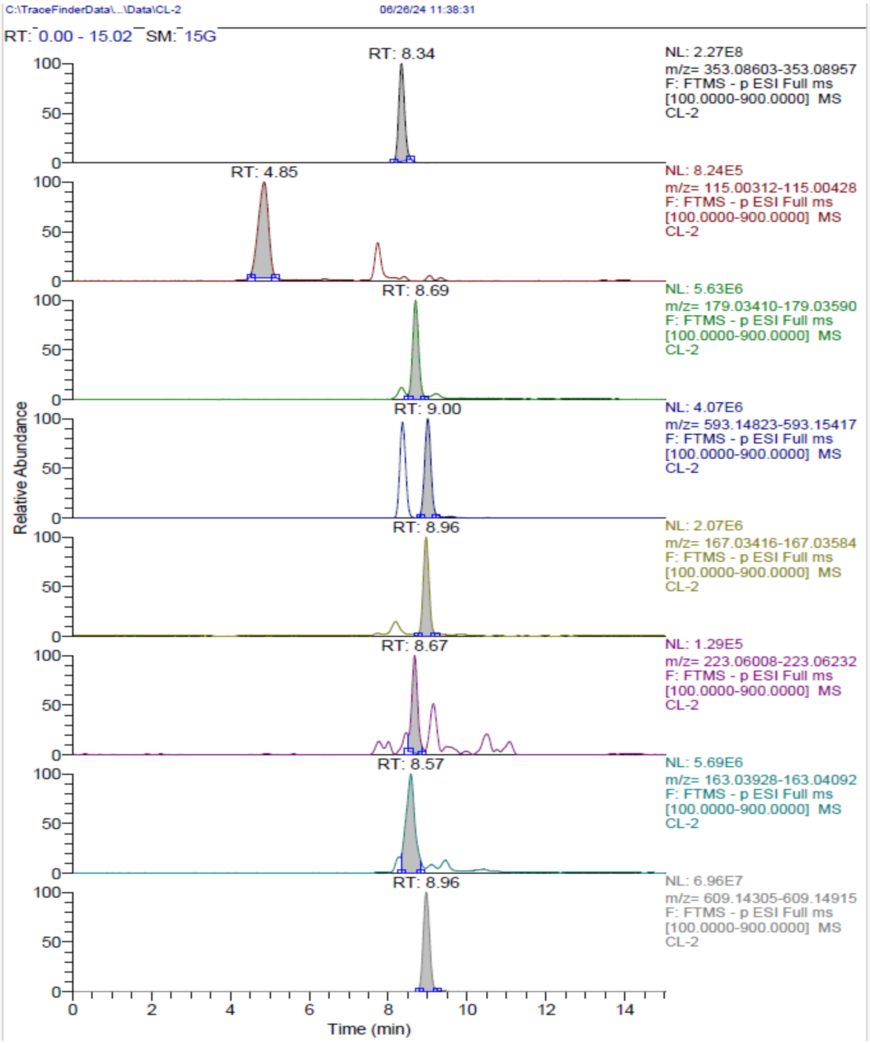

C:\TraceFinderData\...\Data\CL-2
06/26/24 11:38:31
RT: 0.00 - 15.02 SM: 15G
RT: 8.34
NL: 2.27E8
m/z= 353.08603-353.08957
F: FTMS - p ESI Full ms
[100.0000-900.0000] MS
CL-2
RT: 4.85
NL: 8.24E5
m/z= 115.00312-115.00428
F: FTMS - p ESI Full ms
[100.0000-900.0000] MS
CL-2
RT: 8.69
NL: 5.63E6
m/z= 179.03410-179.03590
F: FTMS - p ESI Full ms
[100.0000-900.0000] MS
CL-2
RT: 9.00
NL: 4.07E6
m/z= 593.14823-593.15417
F: FTMS - p ESI Full ms
[100.0000-900.0000] MS
CL-2
RT: 8.96
NL: 2.07E6
m/z= 167.03416-167.03584
F: FTMS - p ESI Full ms
[100.0000-900.0000] MS
CL-2
RT: 8.67
NL: 1.29E5
m/z= 223.06008-223.06232
F: FTMS - p ESI Full ms
[100.0000-900.0000] MS
CL-2
RT: 8.57
NL: 5.69E6
m/z= 163.03928-163.04092
F: FTMS - p ESI Full ms
[100.0000-900.0000] MS
CL-2
RT: 8.96
NL: 6.96E7
m/z= 609.14305-609.14915
F: FTMS - p ESI Full ms
[100.0000-900.0000] MS
CL-2
Relative Abundance
Time (min)

(b)

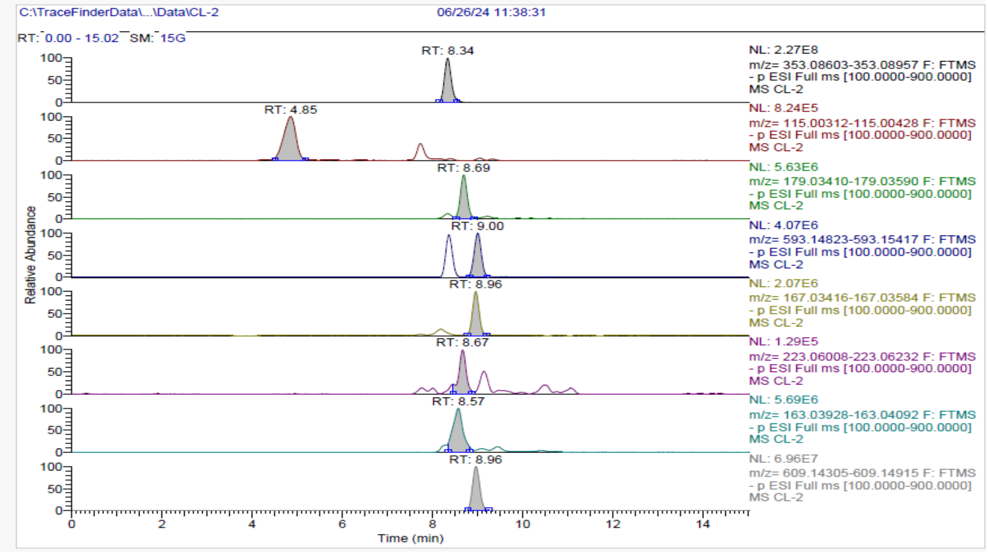

(c)

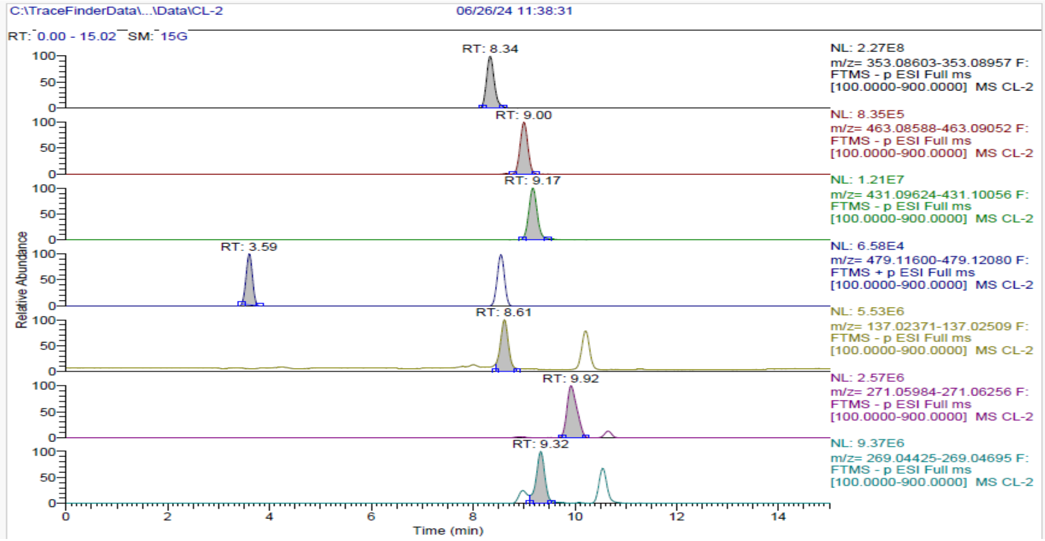

Supplementary Figure 1. (a, b, c); **T**he chromatogram from Phytochemicals' LC-MS (Liquid Chromatography-Mass Spectrometry) analysis of the ethanolic leaf extract of celery (Table 1) shows multiple extracted ion chromatograms (EICs) with different retention times (RT) and corresponding mass-to-charge ratios (m/z).
